# Endoscopic stent treatment of a duodenal ulcer perforation using a semi‐covered stent

**DOI:** 10.1002/ccr3.2293

**Published:** 2019-07-04

**Authors:** Thor Erik Holm, Snorri Olafsson, Airazat M. Kazaryan

**Affiliations:** ^1^ Department of Gastrointestinal Surgery Telemark Hospital Trust Skien Norway; ^2^ Department of Medicine Telemark Hospital Trust Skien Norway; ^3^ Department of Surgery Fonna Hospital Trust Stord Norway; ^4^ Intervention Center Oslo University Hospital ‐ Rikshospitalet Oslo Norway; ^5^ Department of Faculty Surgery N2 I.M.Sechenov First Moscow State Medical University Moscow Russia; ^6^ Department of Surgery N1 Yerevan State Medical University after M.Heratsi Yerevan Armenia

**Keywords:** duodenal ulcer perforation, semi‐covered stent

## Abstract

Placement of a covered self‐expandable metal stent when primary surgery had failed to close the duodenal ulcer perforation or as a primary modality is a promising procedure when compared to other currently available types of surgical rescue or intervention.

## 1 CASE REPORT

**Figure 1:**
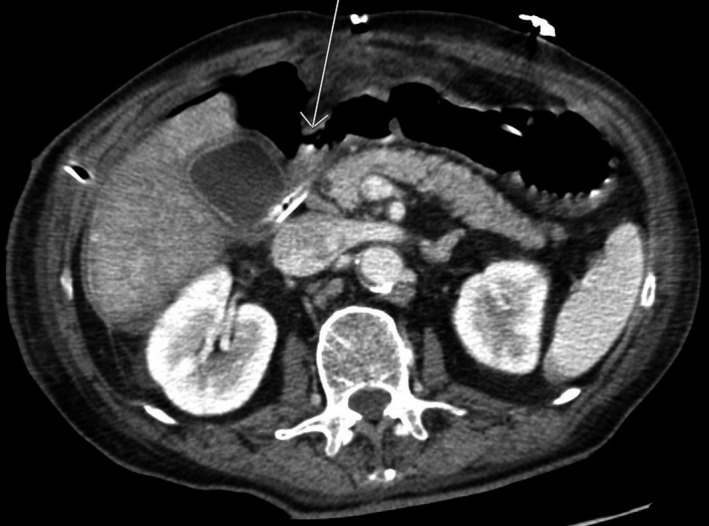
Preoperative computerized tomography. The arrow indicates the ulcer perforation

**Figure 2:**
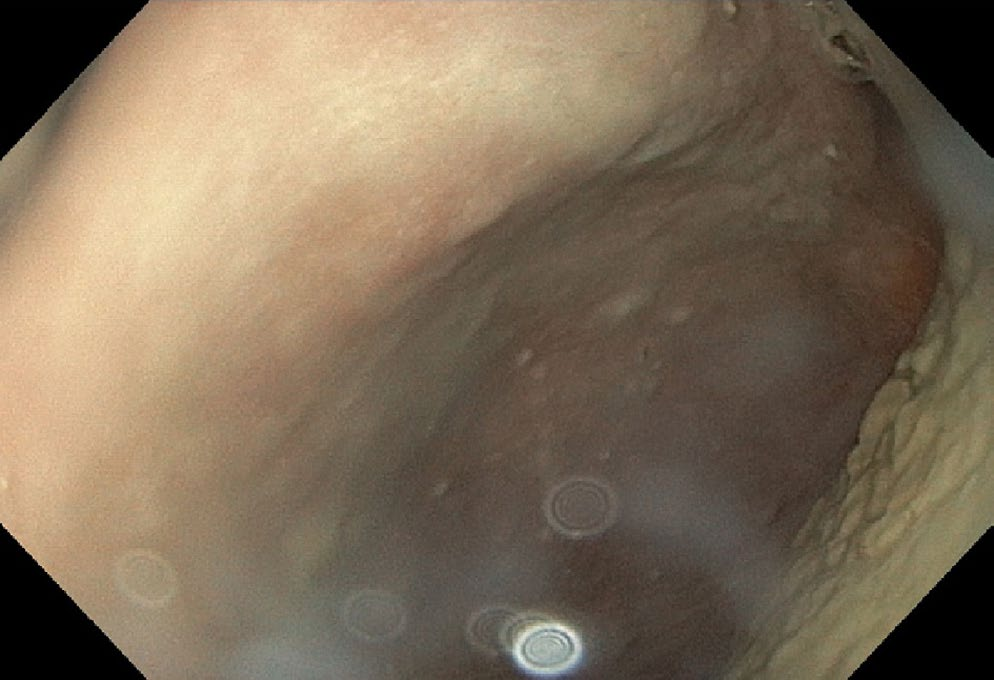
The abdominal cavity visualized through the duodenal perforation with a 5.8 mm upper slim scope

We present a case of a 67‐year‐old man who was admitted with a 4‐day history of abdominal pain. The patient had diffuse peritoneal symptoms and was septic on admission to the hospital. He had earlier been diagnosed with COPD stage 1 and is still smoking around 10 cigarettes a day. He had no other known chronic illnesses and did not use any medication. Broad‐spectrum antibiotics (piperacillin–tazobactam) and intravenous fluid therapy was initiated immediately. A computerized tomography (CT) scan revealed free air in the abdominal cavity (Figure 1). The patient was recently diagnosed with a suspicious lesion in the right lung and a suspected pathological fracture localized in the lumbar spine. He was then operated on utilizing a novel Graham patch for a perforated duodenal ulcer. The perforation was measured at about 1.5 cm in size. An 18 French drain with low active pressure (suction) was left in the abdominal cavity. Postoperatively, there was a persistent leakage of about 1000 mL per day. On the ninth postoperative day, he underwent an upper endoscopy. The perforation was visualized in the duodenal bulb and could easily be traversed with a 5.8 mm upper slim scope (Figure 2).

The patient's clinical condition was tenuous, and it was felt that he would likely not be able to tolerate a major surgical intervention such as a Billroth II reconstruction since he was deemed to be an ASA class 4 according to the American Society of Anesthesiologists. A subsequent consultation with the endoscopic interventional team led to a decision to attempt a stent placement to rescue the failed surgical ulcer closure.

**Figure 3:**
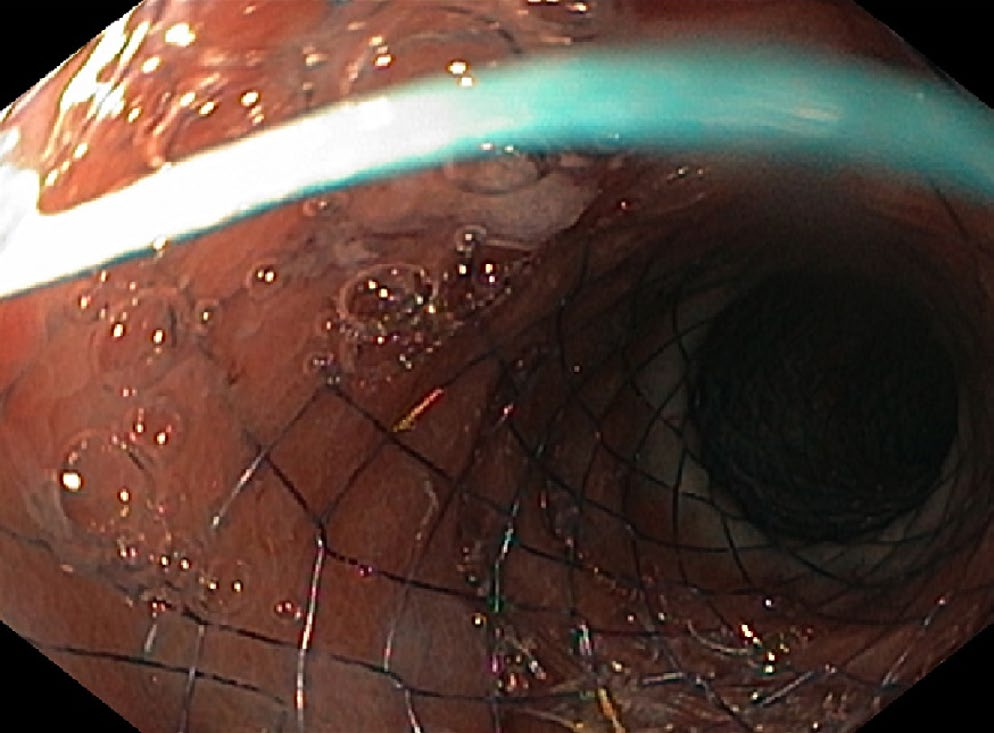
The semi‐covered stent placed in duodenum. Pylorus is visualized in the middle of the picture

Using a therapeutic upper endoscope, we then placed a “through the scope” Hanaro Partially Covered duodenal stent (110 × 26‐20‐26 mm). The covered part of the stent is 74 mm (Figure 3). A good position was confirmed by utilizing fluoroscopy.

**Figure 4:**
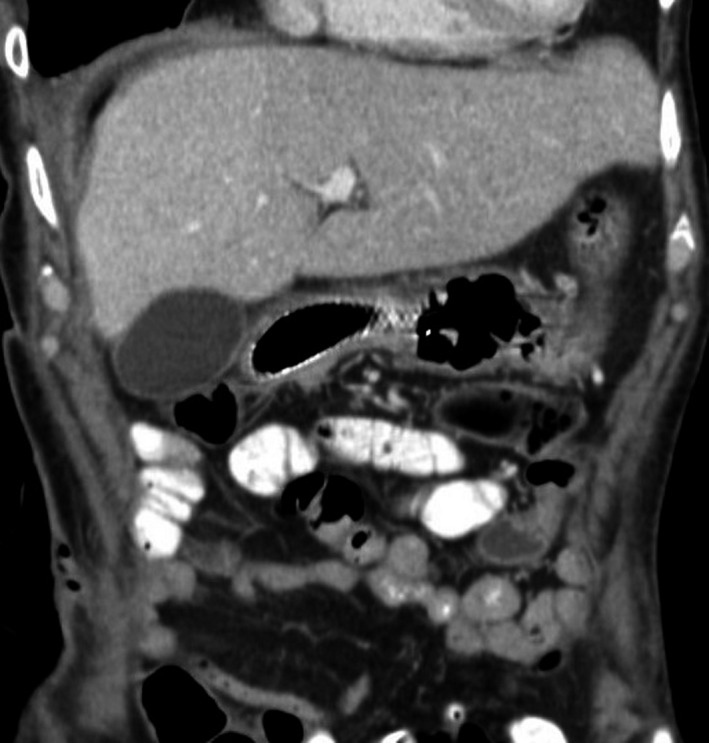
Postprocedural computerized tomography

The general condition of the patient was quite poor, and he had a prolonged recovery. After the endoscopic intervention, the leakage from the drain was reduced considerably in a short period of time. After the stent placement, the patient was given blueberry juice, and there was no evidence of leakage via the drainage catheter. This finding helped confirm that the perforation remained properly closed. The patient was started on an easily digestible diet. The patient was also started on intravenous nutrition within several days. The abdominal drain was removed 10 days after the stent placement. A subsequent CT scan with oral contrast performed 3 weeks after the stent placement showed no leakage into the abdominal cavity (Figure 4). The stent was easily removed after 28 days using gastroscopy with a mild form of anesthesia. The position of the stent was unchanged when it was removed after 4 weeks, which we believe is a sufficient time for the perforation to heal. The patient was discharged from our hospital 2 weeks later with a prescription for oral pantoprazole 20 mg two times daily. Due to the findings of a suspicious lesion in the right lung and a pathologic fracture in the lumbar spine, a subsequent oncologic workup was performed which revealed the presence of lung cancer with metastases to the lumbar spine, the iliac bone, and the left adrenal gland. The patient received palliative radiotherapy. He died 4 months after discharge from the surgical department. The anticipated control gastroscopy was not performed.

## 2 DISCUSSION

Open surgical technique and decades later the laparoscopic closure of a perforated duodenal ulcer represents a standard treatment with reoperation rates below 5%.^1^, ^2^, ^3^

When primary surgery for perforated ulcers fails or when the perforation is accompanied by considerable periduodenal inflammation and is too big for a traditional Graham patch procedure, this usually represents a challenging and complicated procedure. One option includes a larger resection as in a Billroth II procedure, carrying a significant risk of morbidity and mortality.^1^, ^4^, ^5^, ^6^, ^7^ Poor outcomes are typically observed in patients with significant peritonitis and severe comorbidities.^8^ Several technical solutions have been suggested to improve outcomes; however, this has not led to a noticeable improvement in morbidity, relaparotomy, and mortality rates.^5^, ^6^, ^7^, ^8^, ^9^ Endoscopic treatment of gastroduodenal ulcers is considered a promising alternative.^10^, ^11^ Interestingly, a combination of laparoscopy and endoscopy was newly suggested in case a large duodenal ulcers is found to be associated with difficulty in obtaining a sufficient amount of omentum for omental filling using a laparoscopic approach.^9^ Our group has earlier published a case report where an old patient was reoperated twice before the leakage was finally sealed by placing a covered duodenal stent. The treatment was successful but the fully covered Boston Ultraflex 100 × 23 mm stent migrated and caused a small bowel obstruction some months later.^12^ Two years later, Bergstrøm et al^13^ presented eight patients that were successfully treated with a covered self‐expandable metal stent (SEMS) both when primary surgery had failed and as a primary procedure.

Using a covered SEMS when primary surgery for perforated duodenal ulcers fails is not a commonly performed procedure although it is a promising option when compared to other types of rescue surgery. To prevent migration, we used a partially covered stent. The partially covered stent, while still able to migrate, has a lower risk of migration when compared to a fully covered stents, as shown in larger studies where it was used for gastric outlet obstructions.^14^

## CONFLICT OF INTEREST

None declared.

## AUTHORS CONTRIBUTIONS

TEH, AMK: conceived and designed the study. THE: Acquired the data. TEH, SO, AMK: Analyzed and interpreted data. TEH, AMK: Drafted the manuscript. TEH, SO, AMK: Critically revised the manuscript.
